# MiRNA expression analysis from circulating exosomes in Barrett’s esophagus patients with visceral obesity

**DOI:** 10.1038/s41598-025-29379-3

**Published:** 2025-12-12

**Authors:** Mimi Liu, Jing Lv, Yan Cheng, Jun Wang, Dong Liu, Fenrong Chen

**Affiliations:** 1 Department of Gastroenterology, The Second Affiliated Hospital of Xi’an Jiaotong University, No 157 Xi Wu Road, Xi’an, 710004 Shaanxi Province China; 2 Department of Clinical Laboratory, Honghui Hospital, Xi’an Jiaotong University, Xi’an, China; 3 Department of Gastroenterology, Xi’an Hospital of Traditional Chinese Medicine, Xi’an, China

**Keywords:** Visceral obesity, Barrett’s esophagus, Exosomes, MiRNAs, Gastroenterology, Risk factors

## Abstract

Visceral obesity is a recognized risk factor for Barrett’s esophagus (BE). Circulating exosomal mirnas have been identified as potential biomarkers of BE. The aim of this study was to elucidate the characteristics of plasma exosomal miRNA expression in BE patients with or without visceral obesity and to explore its potential regulatory mechanisms in the pathogenesis of the disease. From June 1, 2017 to August 31, 2020, healthy people and BE patients were recruited at center and divided into four groups: Barrett’s esophagus patients with visceral obesity (VOBE), Barrett’s esophagus patients (BE), visceral obese controls (VOC), and healthy controls (HC).Serum samples were collected after approval by the Ethics Committee, exosomes were isolated by ultrafast centrifugation, and the expression of candidate mirnas was verified by qRT-PCR. Data showed that 19 of the common 27 differential miRNAs were up-regulated and two down-regulated among the three comparison groups of VOBE vs. VOC, VOBE vs. HC and VOBE vs. BE.By integrating TargetScan, miRDB and mirDIP databases, 594 target genes were predicted. The enrichment analysis of KEGG pathway suggested that these genes were significantly enriched in the cancer, PI3K-Akt and other pathway. There is an association between some circulating exosomal miRNAs and Barrett’s esophagus in patients with visceral obesity.This provides some ideas for exploring the molecular mechanism of visceral obesity involved in the development of BE.

**Supplementary Information:**

The online version contains supplementary material available at 10.1038/s41598-025-29379-3.

## Introduction

Barrett’s esophagus (BE) is a metaplastic condition, in which the squamous epithelium lining of the lower esophagus is replaced by a columnar epithelium, with or without intestinal metaplasia (IM). BE is a well-accepted risk factor of esophageal adenocarcinoma (EAC). In recent years, the prevalence of BE and EAC is rising constantly, and the incidence of EAC developed from BE patients is much higher than general population^1^. So BE has already become a focus of attention and its pathogenesis need to be revealed. In addition to chronic acid reflux symptoms, age, obesity, smoking, family history of BE or EAC are also considered risk factors for BE and EAC^2^. Obesity, especially visceral obesity, has recently attracted people’s attention. The metabolism disorders induced by visceral obesity is remarkably associated with the development of BE and EAC. However, the association between BE and visceral obesity is still unclear.

Exosomes, which are membrane-derived vesicles, could regulate functions of neighboring cells and cells at distance by means of autocrine, paracrine and endocrine as a new communication pattern among cells. Exosomes could carry and transfer miRNAs into recipient cells, so that miRNAs could play a role in cell biological activities, such as cell proliferation, differentiation, migration and apoptosis. Researchers have already shown that the expression levels of some miRNAs changed in BE and EAC tissues, and the combination of up-regulation of miR-196a and miR-192 and down-regulation of miR-203 could be biomarkers to identify BE from general population^3^. In addition, the expression of miR-29c-3p and miR-193b-5p is different in BE and EAC, and the down-regulation of miR-4485-5p may be a novel marker of disease severity, which may be important for BE formation^4^.MiRNAs can also identify patients at risk of BE progression to dysplasia and cancer^5^.The releasing of exosomes into the extracellular spaces makes their detection in body fluids possible. Researches about miRNA profiling of disease cell-derived and circulating exosomes may provide potent biomarkers through non-invasive blood tests.

Therefore, the abnormal expression of exosomal miRNAs in the circulation from BE patients with or without visceral obesity may give a hint for BE pathogenesis. However, the roles of exosomal miRNAs in the relation between visceral obesity and BE is still blurred, hence we focused on the exosomal miRNAs profiling among BE patients with visceral obesity, BE patients, obese controls and healthy controls in order to investigate the relations between visceral obesity and BE.

## Materials and methods

### Patient recruitment

From 1 June 2017 to 31 August 2020, individuals with BE were recruited at the Gastrointestinal Endoscopy Center in The Second Affiliated Hospital of Xi’an Jiaotong University. The inclusion criteria were: aged between 18 and 60 years; ability to assign the written informed consent. The exclusion criteria were: evidence of lesion or cancer in the alimentary tract; known inflammatory diseases; thyroid disease; prior history of alimentary surgery; severe uncontrolled systematic dysfunction. Healthy volunteers comprised subjects for annual medical examination without any gastroesophageal reflux and no positive findings under endoscopy. Histopathology was conducted to confirm the presence of intestinal metaplasia (IM) without dysplasia and healthy volunteers had no signs of inflammation.

After inclusion and histopathological examination, subjects were divided into four groups, which are BE patients with visceral obesity(VOBE), BE patients(BE), visceral obese controls (VOC) and healthy controls (HC), according to the guidelines of International Life Sciences Institute and World Health Organization^6^. The diagnostic standard of visceral obesity in this study are BMI ≥ 25 kg/m^2^, the waistline ≥ 90 cm for male and ≥ 80 cm for female. The healthy volunteers were recruited from the individuals for physical examination.The age, gender, weight, waist line and BMI of the participants were collected and difference tests were conducted.

### Blood processing, exosomal isolation

Blood was collected into a serum separation tube and then processed by centrifugation performed at 2500 rpm, 4℃ for 15 min. Then serum were stored in enzyme-free Eppendorf tubes at -80℃ before use.

Exosomes were isolated and purified by ultrafiltration centrifugation^7^.Serum aliquots from each subject were thawed on ice, then centrifuged at 2,000×g and 4℃ for 10 min to remove the remaining blood cells and cell debris. Aspirate the supernatant and filter the serum through a 0.22 μm membrane filter (Millipore, USA) to remove the remaining cell debris and large vesicles.

Then The filtrate was transferred to a cell-stirred ultrafiltration instrument with a 100,000 kDa (Millipore, USA) ultrafiltration membrane placed on it, and ultrafiltration was carried out under nitrogen pressure. Exosomes could remain on the ultrafiltration membrane. Resuspend with an appropriate amount of 1× phosphate buffered saline (PBS), and then perform ultrafiltration under nitrogen pressure again. Repeat this process once. The suspension containing exosomes was collected into centrifuge tubes for ultracentrifugation (4℃,100,000×g,1 h). Discard the supernatant, resuspend with 1×PBS, and filter the suspension again using a 0.22 μm membrane filter. The aliquots of exosome suspensions were stored in enzyme-free Eppendorf tubes at -80℃ before use.

### Identification of exosomes

Exosomes were identified by transmission electron microscope, nanoparticle tracking analysis and western blot.Exosome solution(10 µl ) was dropped on the copper mesh, incubated at room temperature for 10 min, and negatively stained with 2% uranyl acetate for 1 min. After drying, the copper mesh was observed under transmission electron microscopy and photographed at 80kv.According to the quantitative results of BCA protein, exosome upper sample size (10–30 µg) was calculated, and protein electrophoresis was performed after denaturation, and the expression of positive exosome proteins including CD63/TSG101/HSP70 and the negative protein marker calnexin were measured by steps.The nanoparticle suspension was irradiated by a laser light source, and the scattered light of the nanoparticle was detected, and the nanoparticle concentration was calculated by counting the number of scattered particles. The exosomes were diluted with PBS to 1 × 10^7^ /ml ~ 1 × 10^9^ /ml, and the size and quality of the exosomes were measured. The particle trajectories of exosomes were also analyzed.

### Ribonucleic acid extraction and quality verification

Extraction of exosomal ribonucleic acid was performed using TRIzol Reagent (Invitrogen Life Technologies, USA) according to the manufacturer’s protocol. The following RNA elution steps included chloroform extraction and isopropanpol extraction. After the final elution with RNase-free ultra pure water, quantity and quality of extracted RNAs have been estimated with Nano Drop 2000 (Thermo Fisher Scientific, USA) phptpmetrically.All qualified samples were used for miRNA chip analysis and follow-up experiments.

### Exosomal MiRNAs array

The GeneChip^®^ miRNA 4.0 Array panel (Affymetrix, USA) was used for miRNAs profiling, which has 100% miRBase v20 coverage. This panel comprised 30,424 mature miRNAs, including 5,214 human, mouse and rat miRNAs and 1,996 human snoRNAs and scaRNAs. It also contains 3,770 probe sets unique to human, mouse and rat pre-miRNA hairpin sequences. RNA poly(A) tailing and FlashTag™ Biotin HSR ligation was applied according to the manufacturer’s instructions from FlashTag™ Biotin HSR RNA Labeling Kit (Affymetrix, USA). After these procedures, the hybridization, washing and staining were conducted to the instructions from GeneChip^®^ Hybridization, Wash and Stain Kit (Affymetrix, USA). Finally, Scanner 3000 (Affymetrix, USA) was used to acquire the scanning results of each miRNA array chip.

### Exosomal MiRNAs data analysis

The microarray data analysis was performed on Transcriptome Analysis Console (TAC) Software (version 4.0, Thermo Fisher Scientific Co.,USA). Differentially expressed miRNAs were determined as miRNAs with a cut-off -value of 2-fold change (FC) and *P* < 0.05.

The clustering hierarchy was used to display the differences in exosomal miRNAs expression patterns between the samples.The target genes of miRNAs were searched by Web tool, including TargetScan 8.0 (http://www.targetscan.org/)^8^, miRDB (https://mirdb.org/)^9^ and mirDIP (http://ophid.utoronto.ca/mirDIP/index.jsp)^10^. In order to study the function and related pathways of target mRNAs, Gene ontology (GO) enrichment analysis and kyoto encyclopedia of genes and genomes (KEGG) analysis were performed using metascape online analysis platform (http://www.metascape.org/)^11^. Venn diagram, enrichment bar chart and dot bubble were plotted by https://www.bioinformatics.com.cn, an online platform for data analysis and visualization.Protein-protein interaction (PPI) networks were built using STRING^12^ (https://www.string-db.org/, Version 12.0) database. After that, the key nodes of the dataset were analyzed and visualized with Cytoscape software and CytoHubba plug-ins.

### MiRNA verification by qRT-PCR

The total RNA extracted from exosomes was detected by qRT-PCR using the Mir-X™ miRNA First-Strand Synthesis reverse transcription kit and the SYBR Premix Ex Taq^TM^ⅡPCR kit (Takara, Japan).All mirnas and U6 (internal standard) primers were provided by Tiangen Biotech. Each sample was repeatedly detected three times, and the data were presented as the relative level of target miRNA expression normalized to U6. Relative expression levels of candidate miRNAs were calculated according to the 2 ^− ΔΔCt^ method.

### Statistical analysis

Statistical analysis was conducted with SPSS software (PASW v18, IBM SPSS, Chicago, IL, USA) and GraphPad Prism software (v5, GraphPad Software, San Diego, CA, USA). Data was displayed as mean ± standard deviation. Quantitative data were obtained using Student’s t tests and one-way anova, and enumeration data were obtained using Fisher’s exact test. *P* < 0.05 were considered significant.

## Results

### Characteristics of subjects

Initially, 45 BE patients were included after endoscopic procedure, 23 of whom were diagnosed as columnar epithelial metaplasia with pathological confirmation. Finally, 22 BE patients and 15 controls without BE were included. After grouping according to the diagnostic criteria for visceral obesity, we finally got VOBE, BE, HC and VOC subgroups described above. There were significant differences in body weight, waist circumference and BMI between the VOC and HC groups, and between the VOBE and BE groups (*p* < 0.05).There was no significant difference in age among the four groups (*p* = 0.9328), and pairwise comparisons of gender in each group showed no difference (*p* > 0.999). Please refer to Table 1 for detailed information.

**Table 1:** Characteristics of subjects in the three groups.

	Healthy controls	Visceral obese controls	Visceral obese Barrett’s esophagus	Barrett’s esophagus	*P* value
Gender (male: female)	4:3	5:3	8:4	6:4	> 0.9999
Mean age, year	51.1 ± 7.2	48.9 ± 6.6	50.9 ± 4.6	50.6 ± 5.7	0.9328
Mean weight, kg	57.79 ± 1.93	84.21 ± 8.79	74.42 ± 6.15	57.5 ± 4.0	4.45 × 10^− 5^ 4.09 × 10^− 5^
Mean waist line, cm	81.6 ± 2.9	101.4 ± 6.1	95.3 ± 4.8	78.4 ± 6.7	1.18 × 10^− 4^ 1.06 × 10^− 5^
Mean BMI, kg/cm^2^	22.01 ± 1.42	28.31 ± 3.08	26.98 ± 2.04	21.63 ± 1.49	2.04 × 10^− 3^ 1.36 × 10^− 5^

Data are the mean ± SD, or the number of patients.

In the tables with two P values, the upper one is the P value compared between the VOC group and the HC group, and the lower one is the P value compared between the VOBE group and the BE group.

### Identification of exosomes

**Fig. 1:**
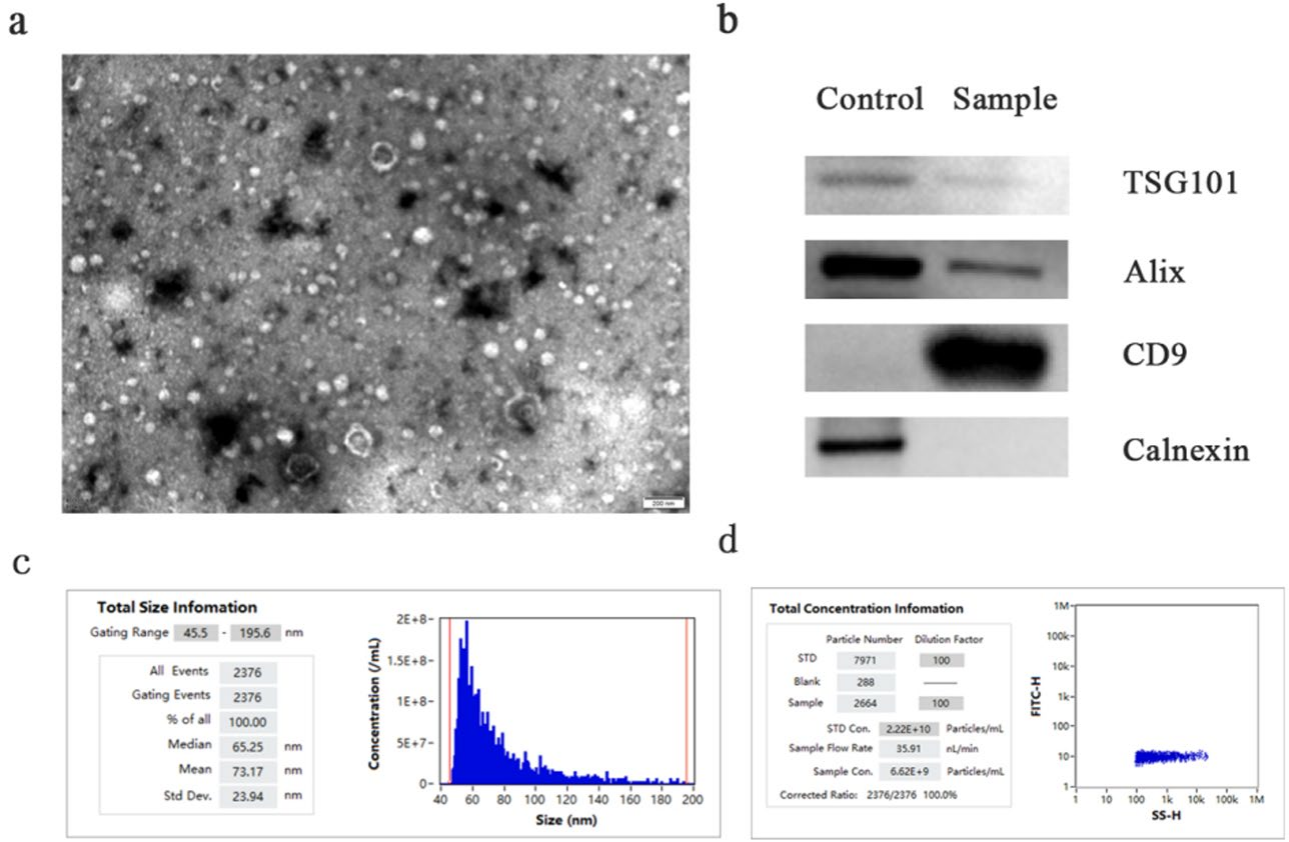
Identification of exosomes. (**a**) Transmission electron microscopy identified morphological characteristics of exosomes. Scale bar: 200 nm. (**b**) Western blot shows the the expression of positive protein markers and negative protein marker. (**c**) Nanoparticle tracking analysis shows the total size of exosomes. (**d**) The total concentration infomation of exosomes.

Transmission electron microscopy showed that the extracellular vesicles were cupped (Fig. 1a). Particle size analysis (NTA) detected that the final volume of exosomes in the sample was 210ul, the average particle size was 73.17 nm, and the particle concentration was 6.62 × 10^9^Particles/mL (Fig. 1c and d), which was consistent with the morphology and size of exosomes.We next validated the purity of our exosome preparations by Western blot analysis. We confirmed the absence of non-exosomal markers (Calnexin) and the presence of proteins known to be positive in exosomes (TSG101, Alix, and CD9) (Fig. 1b).

### Expression profiling of circulating exosomal MiRNAs in VOBE group compared to HC group

**Fig. 2:**
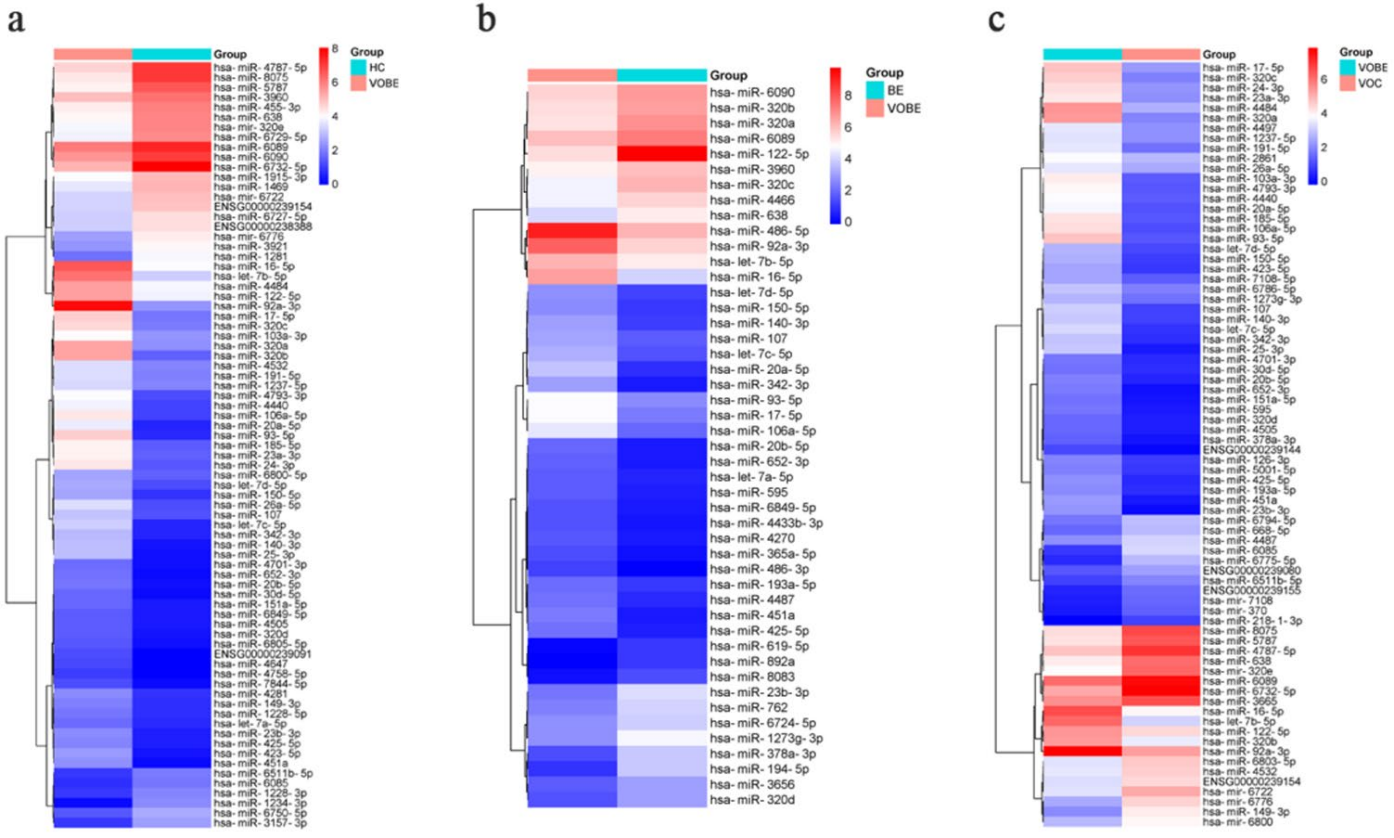
Heat map of differential expression of circulating exosomes between groups. (**a**) VOBE group compared to HC group. (**b**) VOBE group compared to BE group. (**c**) VOBE group compared to VOC group.

Based on TAC analysis, our results showed that there were 78 differentially expressed miRNAs in the comparison group of VOBE vs. HC (Fig. 2a). There were 52 up-regulated miRNAs, and the top five were miR-486-5p, miR-92a-3p, miR-93-5p, miR-320b and miR-17-5p. The first five of the 26 down-regulated miRNAs included miR-1281, miR-4787-5p, hsa-miR-5787,miR-8075,miR-6732-5p.The detailed information was listed in Supplementary Table S1.

### Expression profiling of circulating exosomal MiRNAs in VOBE group compared to BE group

With the same strategy, 48 differentially expressed miRNAs were found in the comparison group of VOBE vs. BE (Fig. 2b). Among the 28 miRNAs up-regulated, miR-92a-3p, miR-149-3p and miR-4532 were the most expressed in cyclically derived exosomes in the VOBE group. On the contrary, the top five of the 20 down-regulated miRNAs were miR-122-5p, miR-194-5p, miR-378a-3p, miR-23b-3p and miR-1273 g-3p.Refer to Supplementary Table S2 for details.

### Expression profiling of circulating exosomal MiRNAs in VOBE group compared to VOC group

In the VOBE vs. VOC group, 77 miRNAs were identified to be differentially expressed (Fig. 2c).There were 51 up-regulated miRNAs, and the top five were miR-486-5p, miR-93-5p, miR-320a, miR-106a-5p, let-7b-5p.The first five of the 26 down-regulated miRNAs included miR-1281, miR-4787-5p, hsa-miR-5787,miR-8075,miR-6732-5p.miR-4787-5p, miR-8075,mir-320e, miR-6085,miR-6732-5p.The detailed information was listed in Supplementary Table S3.

### Differentially expressed MiRNAs in circulating exosomes among VOBE, VOC and HC groups

**Fig. 3:**
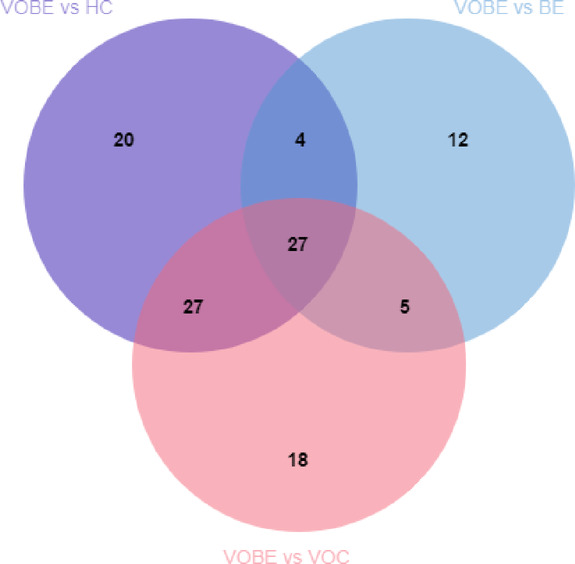
Venn diagram of differential genes in three comparison groups VOBE vs. HC, VOBE vs. BE, and VOBE vs. VOC.

In order to explore the common differential miRNAs among the groups, Venn diagram was made for analysis (Fig. 3). Finally, 27 common differential miRNAs were gathered, among which miR-6089 and miR-638 were down-regulated in all three differential miRNA groups. However, 19 miRNAs were up-regulated in all three different miRNA groups.Six miRNAs were down-regulated in the VOBE vs. BE group and up-regulated in the remaining two differential genomes, namely miR-320b, miR-320d, miR-320a, miR-320c, miR-23b-3p and miR-122-5p.

### KEGG pathway and GO analysis

**Fig. 4:**
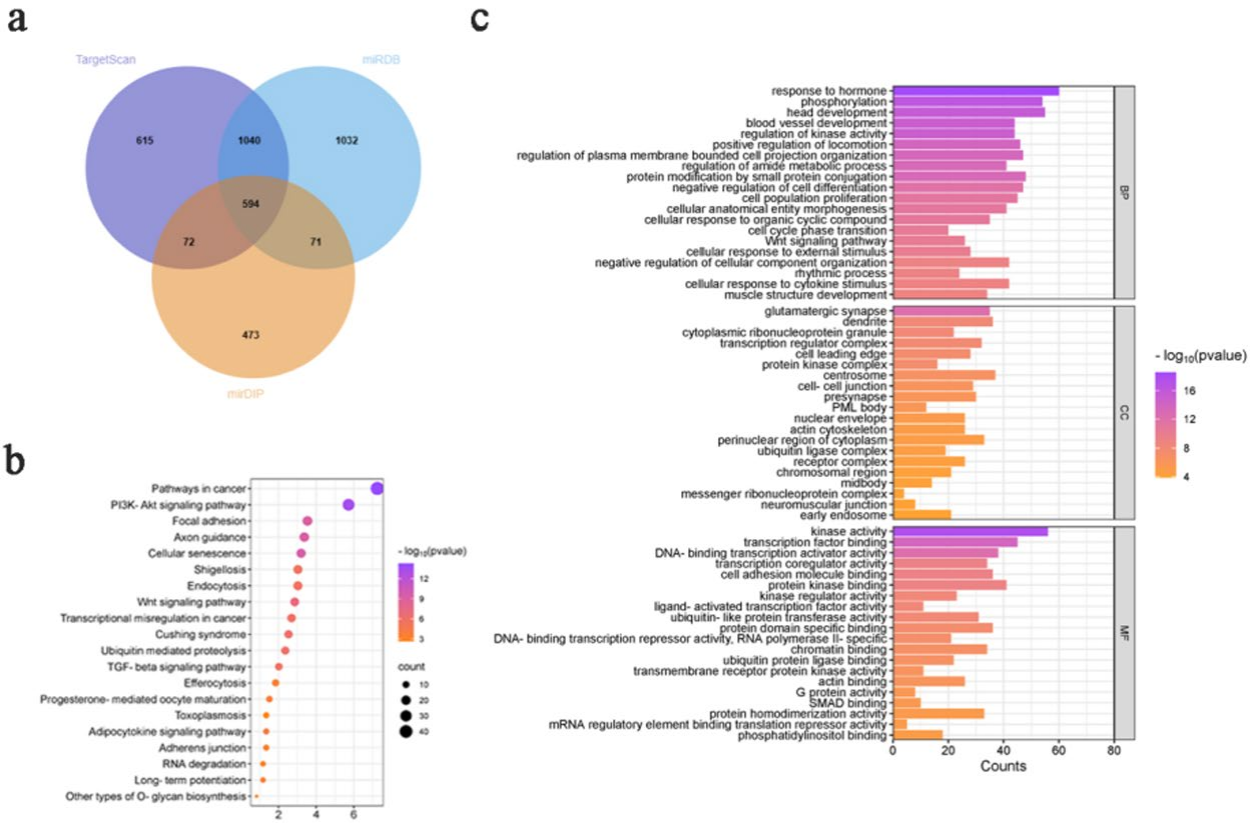
Prediction of differential miRNA target genes and functional analysis. (**a**) Venn diagram of target genes in three databases. (**b**) Bubble maps of the top 20 enriched KEGG pathways. (**c**)Targets of differentially expressed exosomal miRNAs according to the GO analysis.

We obtained 27 potential targets of different miRNAs through TargetScan, miRDB and mirDIP databases, and obtained 594 common target genes by intersection using Venn diagram(Fig. 4a, Table S4).Then, the functions and regulation mechanism of these genes were identified by GO and KEGG enrichment analysis.The pathways in cancer, PI3K-Akt signaling pathway, cellular senescence, axon guidance and focal adhesion represented the top five enriched signaling pathways (Fig. 4b)^13^.GO enrichment analysis included biological process (BP), molecular function (MF), and cell component (CC).As shown in Fig. 4c, the enriched GO functions for target genes in the BP category included the response to hormone, phosphorylation, head development, blood vessel development and regulation of kinase activity.The top five meaningful MF items were kinase activity, transcription factor binding, DNA-binding transcription activator activity, transcription coregulator activity and cell adhesion molecule binding.Regarding CC, the genes were significantly enriched in glutamatergic synapse, dendrite, cytoplasmic ribonucleoprotein granule, transcription regulator complex and cell leading edge.

### PPI network

**Fig. 5:**
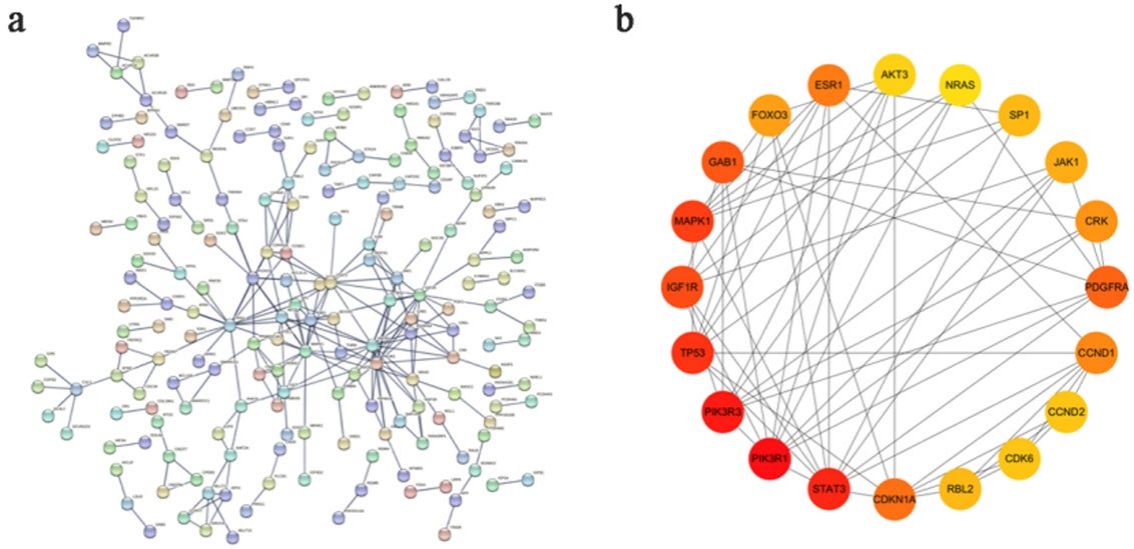
PPI network of target genes. (**a**) PPI network analysis applied in the STRING database. (**b**) Plug-ins CytoHubba of Cytoscape software to screen 20 hub genes.

To screen the key genes to reveal the regulation mechanism of differential miRNAs, the 594 target genes were analyzed with the STRING database and then visualized by Cytoscape software.Twenty core genes were selected by cytoHubba (Fig. 5).

### qRT-PCR validation of randomly selected MiRNAs in circulating exosomes

**Fig. 6:**
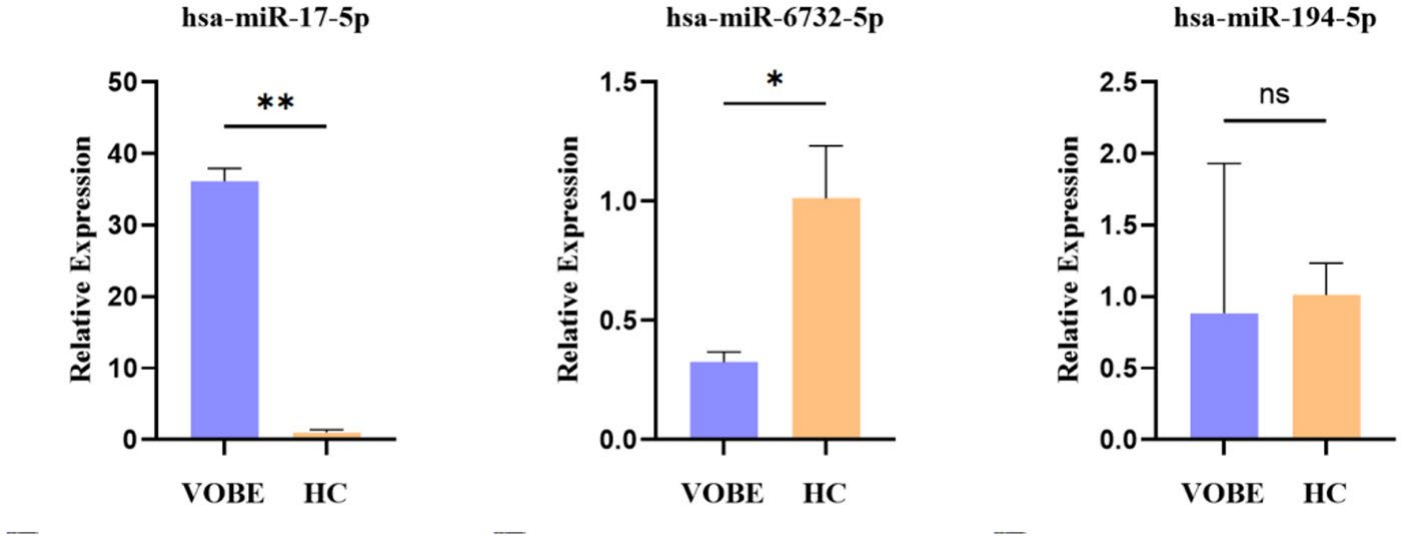
qRT-PCR validation of randomly selected miRNAs in circulating exosomes. After isolation of circulating exosomes, we validated miRNAs profiling results in other samples with randomly selected miRNAs in groups of BE patients with visceral obesity and healthy controls, by qRT-PCR. * and **The expression difference is significant.

Subsequently, three miRNAs were randomly selected to verify the analysis results of the VOBE group and the HC group, including upregulated miR-17-5p (*p* = 0.0013), down-regulated miR-6732-5p (*p* = 0.049) and one non-differentially expressed miRNAs (FC < 2) which was miR-194-5p (*p* = 0.8808). Our results indicated that the microarray data was reliable (Fig. 6).

## Discussion

Molecules performing specific functions are regularly separated and compartmentalized into organelles, and the components in organelles are exchanged dynamically with rest of the cell in order to perform their physiological functions^14^. The majority of organells stay within the cell, while some others such as exosomes are secreted into extracellular spaces. Exosomes contain protein, lipids and nucleic acids as DNA and RNA. It has been shown that exosomes may represent a new way of intercellular communication, not only participate in local environment, but also function distal to their original sites^15^.

Circulating miRNAs are potential biomarkers in disease diagnosis, especially in cancers. They are bound to different carriers, such as proteins, lipoprotein particles and exosomes^16^. And exosomal miRNAs may be more eligible for further analysis. In 2015, Bus Pauline et al.^17^ proposed that circulating miRNAs were differentially expressed in BE and EAC. Chiam Karen et al.^18^ conducted the first study about circulating exosomal serum miRNAs in EAC, BE and healthy controls. The five identified mirnas have diagnostic value for EAC, but their diagnostic value for BE was not elaborated in detail.MiRNAs have been shown to be dys-regulated in BE and EAC. Despite these findings, miRNAs haven’t been implemented in the clinical setting because of conflicting results from different studies.

Obesity, the prominent problem in Western countries, has been taken place in China after the rapid economic development and the change in our lifestyles. Compared with the commonly used measurement method of BMI, waist circumference can be used for a preliminary assessment of visceral fat deposition. The combination of waist line and BMI has been used to explore the correlation of obesity and metabolic disorders or other diseases. Obesity has close relation with various digestive diseases^19^, more than half BE patients accompanied with visceral obesity and visceral obesity could increase the risk of BE by 2 times^20^. Although multiple epidemiological studies and meta-analyses have shown that visceral obesity, characterized by increased abdominal circumference, is more closely associated with the development of BE and its carcinogenicity than BMI^21^, the association and underlying mechanisms still need to be elucidated.

In obese body, the numbers of adipocytes are increased significantly and the adipocytes could secrete specific exosomes, which may affect the pathogenesis of related disease^15^. Ortega FJ et al.^22^ detected circulating mirnas in obese subjects after weight loss and found that miR-15a, miR-520c-3p and miR-423-5p were specific for morbidly patients with obesity. Ferrante SC et al.^23^ also raised that 55 miRNAs derived from adipocytes were differentially expressed between obese and lean subjects. Overall, circulating miRNAs are dys-regulated in obese subjects. More studies focusing on the effects of dys-regulated circulating miRNAs, and it is necessary to provide further insights into the specific role of miRNAs in obesity-associated diseases.

Therefore, we focused on whether visceral obesity plays an important role on BE pathogenesis by means of exosomes. In this study, we In this study, we analyzed the miRNAs spectra from circulating exosomes. After intersection acquirement, we found that 19 miRNAs were the up-regulated miRNAs among the three comparison groups of VOBE vs. VOC, VOBE vs. HC and VOBE vs. BE, and the higher expression were miR-486-5p, miR-93-5p, miR-106a-5p, miR-92a-3p and miR-25-3p. However, miR-6089 and miR-638 were both down-regulated in the three differential miRNA groups. The results suggest that visceral obesity-related circulating exosomes may play an important role in the pathogenesis of BE.

KEGG and GO enrichment analysis of 27 differentially predicted miRNA targets showed that obesity-related miRNAs promoted the development of Barrett’s esophagus mainly through the PI3K-Akt signaling pathway, Wnt signaling pathway and TGF-beta signaling pathway. Among them, the role of Wnt signaling pathway activation in Barrett’s esophagus has been extensively studied^24^.The expression of TGF-β was lower in the tissue of BE^25^, which is known to have tumor-suppressive activity across gastrointestinal tumors, whereas once the TGF-β pathway is deregulated, it could have tumor-promoting activity^26^. In addition, conjugated bile acids can induce the expression of Barrett’s esophagus marker MUC5AC by activating the PI3K-Akt signaling pathway^27^, but whether the promotion of Barrett’s esophagus by this pathway is related to obesity has not been reported.

There have been studies on the five highly expressed differential mirnas screened out above in obesity-related diseases and cancer.Plasma miR-486-5p concentration was significantly increased in children with obesity, and was positively correlated with body mass index, waist circumference and other obesity indicators^28^. A meta-analysis of differential miRNA expression after bariatric surgery showed that 12 mirnas, including miR-93-5p and miR-92a-3p, were down-regulated after surgery^29^. The exosome miR-25-3p in saliva was significantly enriched in obese type 2 diabetes patients, which contributed to the development of diabetes-related periodontitis^30^.

Emerging evidence has revealed that miR-486-5p acts as an oncogene in various cancers.The high expression of miR-486-5p in CD31 vascular endothelial cells increased vascular permeability and promoted NSCLC metastasis^31^. Several studies indicated that cancer associated fibroblasts transfer the exosome miR-92a-3p into colorectal cancer cells and activate the Wnt/ β-catenin pathway, promoting the stemness, EMT, and metastasis of colorectal cancer cells^32^. Circulating miR-92a-3p may also be a novel biomarker of Barrett’s carcinogenesis^33^. The miR-93-5p promotes the proliferation, migration and invasion of EC cells and inhibits apoptosis by targeting TGFβR2^34^. MiR-93-5p is also a biomarker for a variety of tumors, including bladder cancer^35^, breast cancer^36^ and prostate cancer^37^. Similarly, miR-106a-5p can also be used as a potential marker for breast cancer^36^, gastric cancer^38^, lung squamous cell carcinoma^39^ and other tumors.

Nevertheless, the role of circulating exosomal MiRNA in Barrett’s esophagus is unclear.An earlier study found that microRNA could be used as a biomarker for Barrett esophageal progression, with the sensitivity and specificity of miR-486-5p for HGD / EAC being 82 and 55%, respectively^40^.A recent study found that the expression of miR-92a-3p increased gradually in normal squamous epithelium, Barrett’s mucosa, high-grade dysplasia and barrett adenocarcinoma, suggesting that miR-92a-3p may be a novel biomarker of Barrett’s carcinogenic effect^41^.Although our results may provide a new perspective on the pathogenesis of BE, the role of obesity-related mirnas such as miR-486-5p and miR-92a-3p in the pathogenesis of BE needs further research to explore the relationship between visceral obesity and BE.

There are still some limitations in this study. Firstly, this is a single-center study with a small sample size, which is insufficient to draw the final conclusion of clinical application. The sample size should be expanded for further correlation verification. Secondly, although we have observed that the expression of exosome mirnas is associated with Barrett’s esophagus, there is a lack of key experimental evidence. Basic research on the mirnas of interest is needed to explore the specific molecular mechanisms. Furthermore, when choosing mirnas for PCR verification, only randomness was considered and the significance of differences was ignored. Based on these limitations, the current research results are more suitable for preliminary exploration. More experiments are needed to verify the application of circulating exosome miRNAs detection in the risk prediction and diagnosis of be, and to conduct large-scale cohort studies as an invasive marker.

## Conclusion

In summary, our research results have revealed the correlation between the specific exosomal miRNA profiles of individuals with visceral obesity and Barrett’s esophagus. Further functional validation is required to establish whether these miRNAs have a direct role in disease development.

## Supplementary Information

Below is the link to the electronic supplementary material.

Supplementary Material 1

Supplementary Material 2

## Data Availability

The datasets used and/or analysed during the current study available from the corresponding author on reasonable request.

## Abbreviations

BE: Barrett’s esophagus
EAC: Esophageal adenocarcinoma
IM: Intestinal metaplasia
PBS: Phosphate buffered saline
GO: Gene ontology
KEGG: Kyoto encyclopedia of genes and genomes
PPI: Protein-protein interaction
BP: Biological process
MF: Molecular function
CC: Cell component
