# Supplementary figures and images for: MiRNA expression analysis from circulating exosomes in Barrett’s esophagus patients with visceral obesity
Source: Sci Rep. 2025 Dec 12;16:219. doi: 10.1038/s41598-025-29379-3 (PMC12770631; doi:10.1038/s41598-025-29379-3)

## Uncropped Western blot

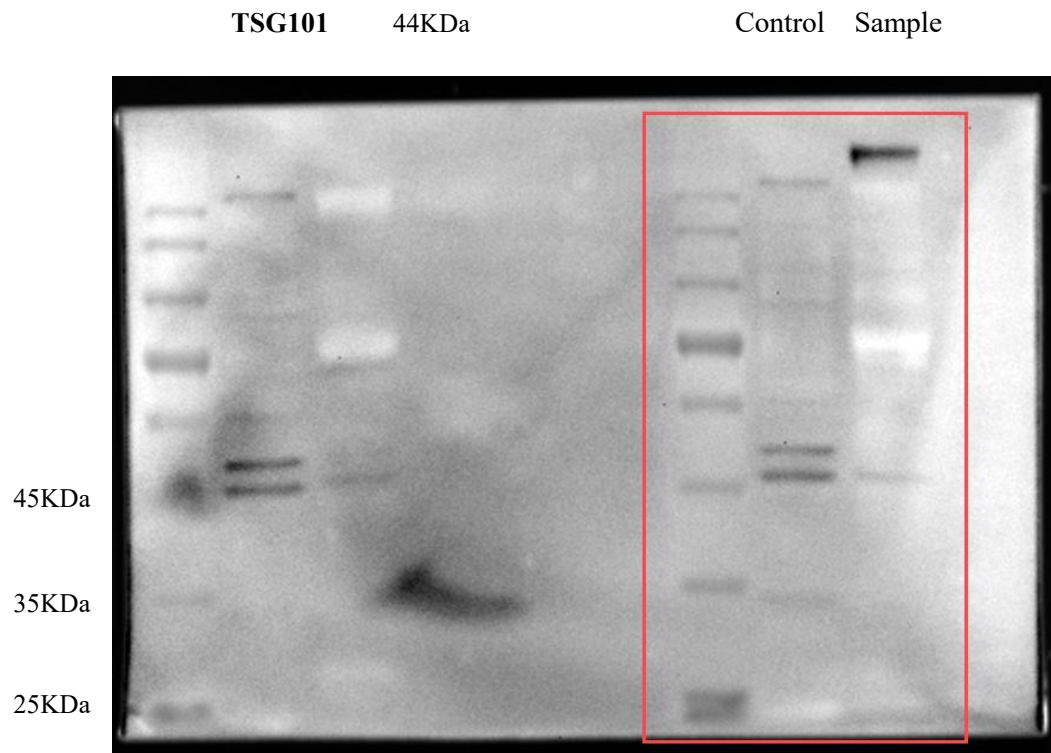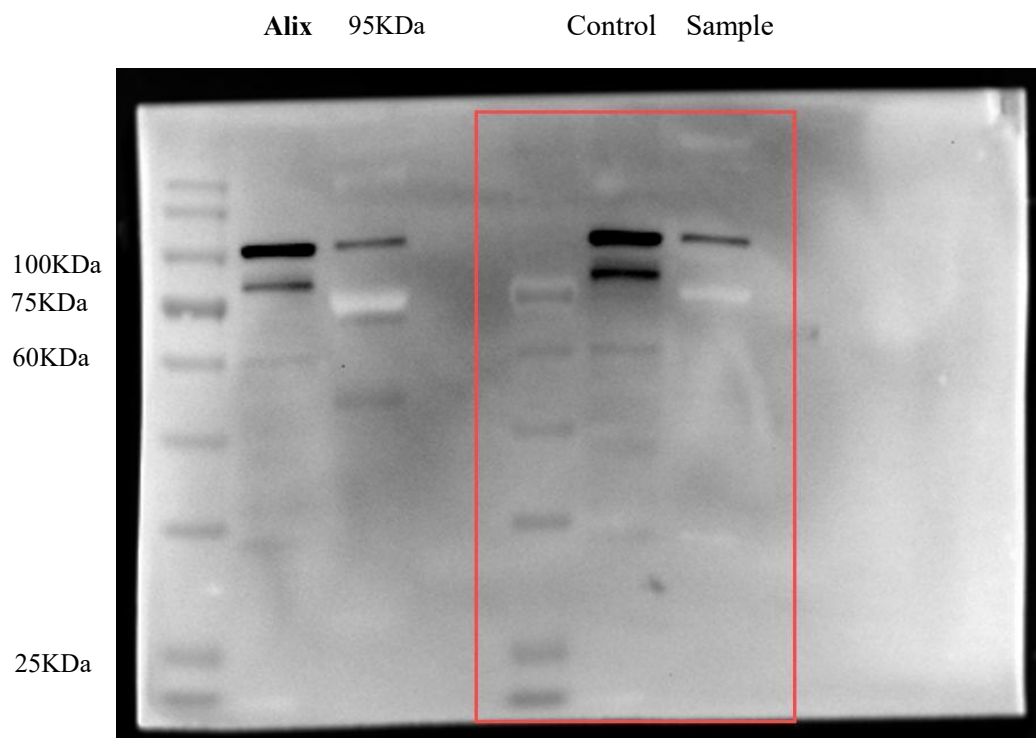

**CD9** 24KDa

**Control** **Sample**

25KDa

15KDa

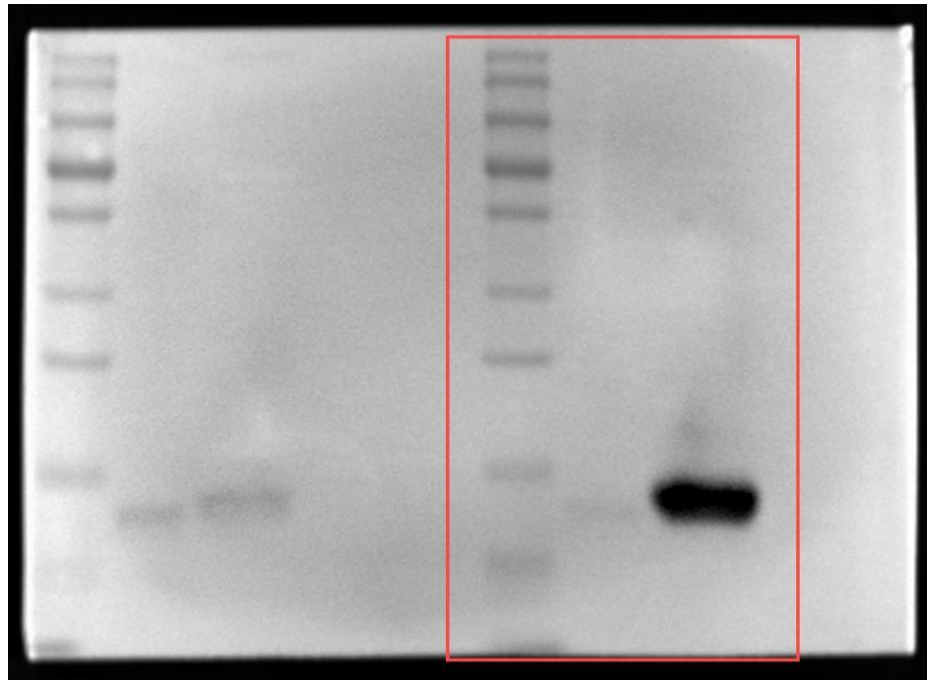

**Calnexin** 90KDa

**Control** **Sample**

100KDa

75KDa

25KDa

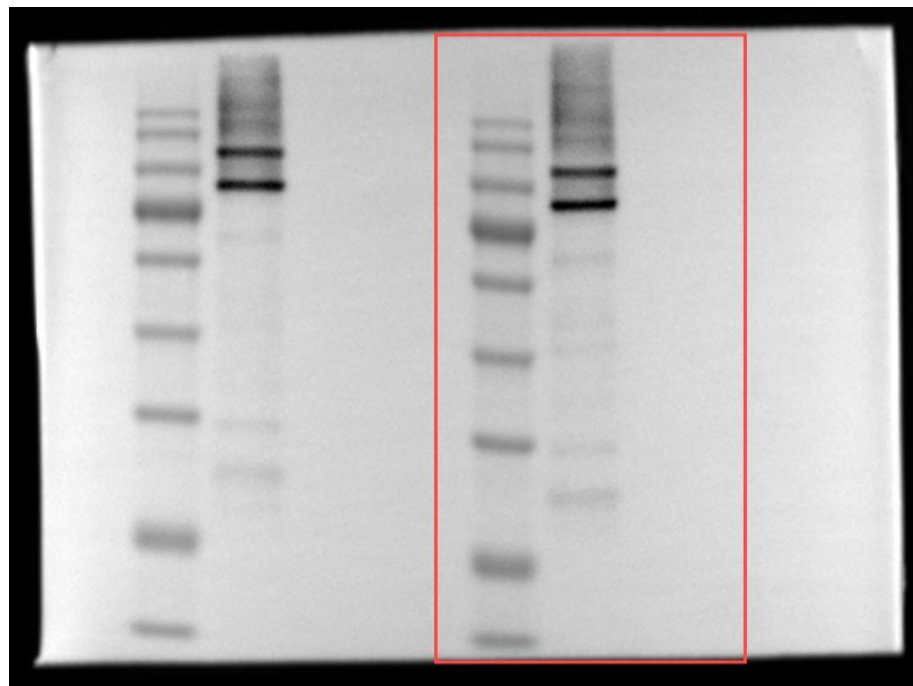

Supplement: Supplementary file 1 — Supplementary Material 1 [file 41598_2025_29379_MOESM1_ESM.pdf]
